# Wnt/β-Catenin Pathway Regulates Cementogenic Differentiation of Adipose Tissue-Deprived Stem Cells in Dental Follicle Cell-Conditioned Medium

**DOI:** 10.1371/journal.pone.0093364

**Published:** 2014-05-07

**Authors:** Na Liu, Bin Gu, Ning Liu, Xin Nie, Bo Zhang, Xia Zhou, Manjing Deng

**Affiliations:** 1 Department of Stomatology, Chinese PLA General Hospital, Beijing, China; 2 Department of Stomatology, Chinese PLA 15 Hospital, Lanzhou, China; 3 Department of Stomatology, Institute of Surgery Research, Daping Hospital, The Third Military Medical University, Chongqing, China; 4 State Key Laboratory of Trauma, Burns and Combined Injury, Institute of Surgery Research, Daping Hospital, The Third Military Medical University, Chongqing, China; 5 Department of Stomatology, Institute of Surgery Research, Daping Hospital, The Third Military Medical University, Chongqing, China; University of Udine, Italy

## Abstract

The formation and attachment of new cementum is crucial for periodontium regeneration. Tissue engineering is currently explored to achieve complete, reliable and reproducible regeneration of the periodontium. The capacity of multipotency and self-renewal makes adipose tissue-deprived stem cells (ADSCs) an excellent cell source for tissue regeneration and repair. After rat ADSCs were cultured in dental follicle cell-conditioned medium (DFC-CM) supplemented with DKK-1, an inhibitor of the Wnt pathway, followed by 7 days of induction, they exhibited several phenotypic characteristics of cementoblast lineages, as indicated by upregulated expression levels of CAP, ALP, BSP and OPN mRNA, and accelerated expression of BSP and CAP proteins. The Wnt/β-catenin signaling pathway controls differentiation of stem cells by regulating the expression of target genes. Cementoblasts share phenotypical features with osteoblasts. In this study, we demonstrated that culturing ADSCs in DFC-CM supplemented with DKK-1 results in inhibition of β-catenin nuclear translocation and down-regulates TCF-4 and LEF-1 mRNA expression levels. We also found that DKK-1 could promote cementogenic differentiation of ADSCs, which was evident by the up-regulation of CAP, ALP, BSP and OPN gene expressions. On the other hand, culturing ADSCs in DFC-CM supplemented with 100 ng/mL Wnt3a, which activates the Wnt/β-catenin pathway, abrogated this effect. Taken together, our study indicates that the Wnt/β-catenin signaling pathway plays an important role in regulating cementogenic differentiation of ADSCs cultured in DFC-CM. These results raise the possibility of using ADSCs for periodontal regeneration by modifying the Wnt/β-catenin pathway.

## Introduction

Periodontitis is one of the most common dental infectious diseases, in which inflammation extends deep into the tissues, thus damaging the periodontal tissue composed of periodont ligament, gingiva, alveolar bone and cementum covering the tooth root. In advanced cases, the destruction of periodontal ligament tissue and alveolar bone may result in tooth loss [1]. The ultimate goal of periodontal therapy is the regeneration of the original architecture and function of the multiple and complex tissues that comprise the periodontium. Periodontal regeneration requires new connective tissue formation and attachment to the root surface, a process that involves the regeneration of periodontal fibers and the insertion of these fibers into the newly formed cementum and bone. Cementum is a calcified connective tissue covering the tooth root surface that, in conjunction with the periodontal ligament and the alveolar bone, forms a rigid tooth-anchoring structure in periodontal tissue [2]. Therefore, cementogenesis is a critical process for the homeostasis and regeneration of the periodontium.

To date, adipose tissue-derived stem cells (ADSCs) have been extensively used in tissue engineering [3]–[5]. Manipulating culture conditions and the cells' microenvironment to direct desired cell lineage differentiation has also been studied extensively. It has been proposed that intercellular communication through growth factors and extracellular matrix (ECM) is the basis for advancing differentiation of adult mesenchymal stem cells into periodontal cells (including cementoblasts, fibroblasts and osteoblasts) [6], [7]. As a preliminary attempt to recapitulate events involved in early cementogenesis, our group could demonstrate that dental follicle cell-conditioned medium (DFC-CM), which likely contains multiple molecular signals and growth factors necessary for ADSC proliferation and differentiation, is able to provide the cementogenic microenvironment and to induce differentiation of ADSCs along the cementoblast lineage [8]. For this to occur, concerted and sequential spatial and temporal events must take place, involving signaling molecules, soluble and insoluble ECMs, and responding stem cells. However, the molecular mechanisms that regulate these processes are still somewhat obscure.

The canonical Wnt/β-catenin pathway stabilizes β-catenin; on activation of the canonical Wnt pathway, β-catenin binds to the T-cell factor/lymphoid enhancer-binding factor (TCF/LEF) transcription factors and mediates the transcription of Wnt target genes [9]–[11]. The Wnt signaling pathway plays an important role not only in embryonic development but also in the maintenance and differentiation of the stem cells [12]–[15]. In particular, Wnt signaling has been shown as an important regulatory pathway in the osteogenic differentiation of mesenchymal stem cells. Dentin and cementum share many similarities with bone in their biochemical compositions and biomechanical properties. A recent study showed that Wnt/β-catenin over-activation during tooth morphogenesis is sufficient to cause dramatic alterations in the adult tooth by delaying cellular differentiation and stimulating proliferation of the dental mesenchyme of developing teeth [16]. Epiprofin/Specificity Protein 6 (Epfn) is a Krüppel-like family (KLF) transcription factor that is critically involved in tooth morphogenesis and dental cell differentiation. Epfn enhances canonical Wnt/β-catenin signaling in the developing dental pulp mesenchyme and regulates Wnt-BMP signaling and the establishment of cellular junctions during the bell stage of tooth development [17]. Scheller et al. indicated that Wnt/β-catenin signaling negatively regulates the odontoblast-like differentiation of dental pulp stem cells [18].

Wnt signaling acting on cementoblasts could inhibit cell differentiation while stimulating cell proliferation [19]. Cho et al. found that the Wnt/β-catenin pathway promoted proliferation and suppressed osteogenic differentiation of human adipose-derived MSCs [20]. A recent study found that by 2 months after implantation, ADSCs incubated with platelet-rich plasma developed into periodontal tissue with the correct architecture, including alveolar bone, cementum-like structures and periodontal ligament-like structures [21]. We speculated that the Wnt signaling pathway could affect the differentiation potential of ADSCs induced by a DFC-CM microenvironment. Thus, we aimed to better define how Wnt influences ADSC commitment along cementoblast-like cell differentiation as well as the mechanisms involved.

## Materials and Methods

All animal experiments were approved by the Animal Care and Use Committee of Third Military Medical University and were carried out in compliance with the “Guide for the Care and Use of Laboratory Animals” published by the National Institutes of Health. All surgery was performed under sodium pentobarbital anesthesia, and all efforts were made to minimize suffering.

### Animals

Harlan Sprague–Dawley (SD) filial rats (7 days old) were used for ADSC culture. SD rats were obtained from the Laboratory Animal Research Centre of the Third Military Medical University, China, and maintained on a daily diet of Purina rodent chow in housing quarters with a light:dark cycle (12 h L∶D), regulated temperature, and sterile water.

### Cell culture and identification

ADSCs were isolated and cultured using a previously described method [3]. Isolated adipose tissues were finely minced into 1×1 mm^3^ small pieces, then incubated in 60 units/mL type I collagenase (Gibco BRL) for 60 min at 37°C to partially dissociate adipose tissues. Single cell-derived colony cultures were obtained using the limiting dilution technique as previously described [22]. After 2–3 weeks in culture, the single cell-derived clones were harvested and mixed together. To assess colony-forming efficiency, subconfluent cultures of ADSCs were fixed with 70% ethanol and then stained with 0.1% Crystal Violet [22]. Then, fluorescence-activated cell sorting (FACS) analysis was used for analyzing STRO-1, CD146, CD31, CD90, CD105, CD34 and CD45 (eBioscience, San Diego, CA, USA). Cells in passage three were cultured in osteoblastic conditions containing L-ascorbate-2-phosphate, dexamethasone, and inorganic phosphate for 21 days. Alizarin Red S (Sigma, St. Louis, MO, USA) staining was performed to determine mineralization. For adipocyte differentiation, cells were incubated for 3 days in adipogenic induction medium (DMEM) with 10% FCS, 1 µM dexamethazone, 10 µM insulin, 0.5 mM 3-isobutyl-1-methyl-xanthine (IBMX) and 100 nM indomethacin) followed by 24 h in maintenance medium DMEM with high glucose, 10% fetal bovine serum (FBS, Hyclone, USA) and 10 µM insulin) (Sigma). This regime comprised one cycle of induction and maintenance. After 21 days of culture, cells were characterized for formation of oil globules by Oil Red O staining.

### Preparation of dental follicle cell-conditioned medium (DFC-CM)

Rat DFCs were isolated and cultured as described previously [8]. Briefly, dental follicle tissue was separated from the first molars under a dissecting microscope. The dental follicles were aseptically dissected and placed in phosphate buffered solution (PBS). The tissue blocks were incubated in DMEM supplemented with 10% FBS in a humidified atmosphere at 37°C and 5% CO_2_. The isolated cells from the DF(dental follicle) grew to confluence in about 2 weeks and remained fibroblastic in shape. No contamination of epidermal cells was observed after two passages. Once the cells reached confluence, culture medium was changed every day and collected for 3 days. The collected media were filtered through a 0.2 µm Millipore strainer and mixed with an equal volume of fresh DMEM supplemented with 10% FBS, then stored at −80°C before used as dental follicle cell-conditioned media.

### Wnt3a or DKK-1 treatment

The primary group of cultures was treated with 100 ng/mL recombinant Wnt3a (R&D Systems) [23], and a subset of these cultures was treated with the soluble Wnt inhibitor DKK-1 (recombinant DKK-1; Peprotech) at 100 ng/mL [24]. Cells were seeded at a density of 5000 cells/cm^2^ in the culture flasks, maintained and expanded in DMEM (10% FBS), and allowed to adhere overnight. The culture medium was then changed to DFC-CM, which contained either Wnt3a or DKK-1 used for the ADSC cultures. On culture day 7, the cells were harvested and subjected to assays for *in vitro* cementoblast-like differentiation.

### Immunohistochemistry

ADSCs were seeded into a 24-well plate (5×10^3^ cells/well) and cultured in basic medium (DMEM plus 10% FBS) for 1 day. Then, the cells were incubated with DFC-CM for another 7 days. They were then fixed in 4% paraformaldehyde for 15 min, after which the cells were incubated with β-catenin (Abcam,32572), CAP (cemetum attachment protein) or BSP (bone sialoprotein) at dilutions ranging from 1∶100 to 1∶200 (Santa Cruz Biotechnology, Inc., Santa Cruz, CA, USA) for 2 h and subsequently incubated with FITC(Fluorescein Isothiocyanate)- or Rhodamine-conjugated anti-rabbit or anti- goat secondary antibodies. Then, sections were counterstained with Hoechst 33342 (5 µg/mL; Sigma) to identify all nuclei. The image collection and superimposition were processed by DP controller (Olympus Optical, Tokyo, Japan) and DP manager (Olympus Optical, Tokyo, Japan). Isotype-matched control antibodies were used under the same conditions.

### Real-time PCR analysis

Total RNA was isolated from cultured cells using the Trizol reagent (Invitrogen). Approximately 2–5 mg of total RNA was converted to cDNA using the Super Script First Strand Synthesis Kit (Invitrogen). The real-time PCR reactions were performed using the QuantiTect SYBR Green PCR Kit (Toyobo, Osaka, Japan) and the Applied Biosystems 7500 Real-Time PCR Detection System. Two independent experiments were performed for each reaction in triplicates. The primers used are listed in Table 1.

**Table 1:** Primer sequences.

Gene	Primer Sequence
CAP	forward:5′-TCTGACGACTCTGCTTCACG-3′reverse:5′- TTCAGGGCATGTGTGATGCT -3′
ALP	forward:5′-ATGTCAACCGAAACGCCTCAG-3′reverse:5′- ATGGCGGAGTCGAACATGGCA-3′
BSP	forward:5′-ACAGCTGACGCGGGAAAGTTG-3′reverse:5′-ACCTGCTCATTTTCATCCACTTC-3′
OPN	forward:5′-AGACCATGCAGAGAGCGAG-3′reverse:5′- ACGTCTGCTTGTGTGCTGG-3′
LEF-1	forward:5′-GGCCCCTCCTACTCCAGTTA-3′reverse:5′- TAGACATGCCTTGCTTGGGG-3′
TCF-4	forward:5′-ATGCATGGGATCATCGGACC-3′reverse:5′-GGTCAGCCAACTCGTCACAGTC-3′
β-ACTIN	forward:5′-CCCTGAAGTACCCCATTGAA-3′reverse:5′-CTAAGTCATAGTCCGCCTAGAAGCA -3′

### Western blot analysis

ADSCs were harvested in a RIPA lysis buffer (P0013B, Beyotime Co., Shanghai, China). After whole cell protein extracts were quantified by a BCA assay, they were separated on NuPAGE 10%–12% polyacrylamide gels, transferred to PVDF membranes (Millipore, Billercia, MA, USA), blocked in 5% BSA in TBST, and hybridized with antibodies against primary mouse β-actin/GAPDH antibody (1∶2000, Abcam, Cambridge, UK), primary rabbit β-catenin (1∶800, Abcam, Cambridge, UK), primary rabbit GSK3β antibody (1∶800, Cambridge, UK), primary mouse CAP antibody (1∶800, Santa Cruz, CA, USA), primary mouse BSP antibody (1∶1000, Santa Cruz, CA, USA), and primary rabbit OPN antibody (1∶1000, Santa Cruz, CA, USA), respectively. Beta-actin or GAPDH on the same membrane were used as a loading control. Signals were revealed after incubation with anti-rabbit or anti-mouse IgG secondary antibody (1∶2000) coupled to peroxidase by using ECL. Densitometry of Western blots was analyzed with Quantity One software and normalized to respective loading control signal on each blot.

### Statistical analysis

Data are presented as mean ± SD. Comparisons were made using a t-test or one-way ANOVA for experiments with more than three groups. All experiments were repeated three times, and representative experiments are shown. Differences were considered significant at *P*<0.05.

## Results

### Biological characteristics of ADSCs

**Figure 1:**
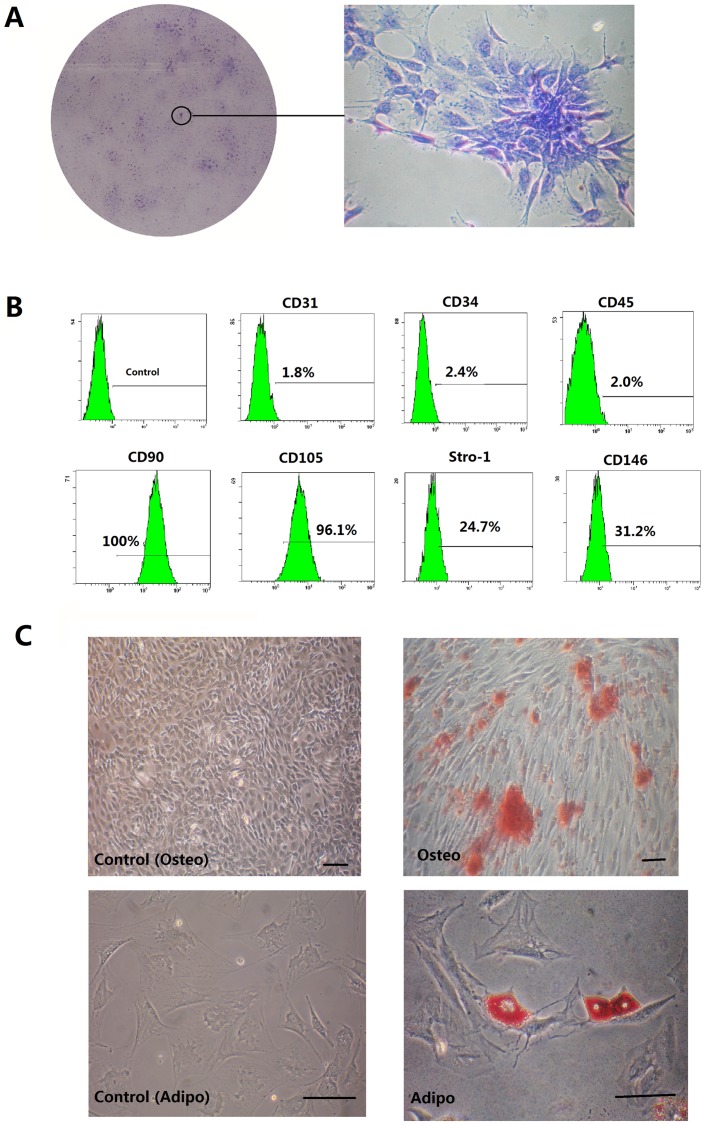
Isolation and identification of ADSCs. (**A**) Representative images of colonies formed by ADSCs at low seeding density after 2 weeks in culture. (**B**). Flow cytometry analysis of the expression of cell surface markers related to mesenchymal (CD31, CD90, CD105, CD146 and STRO-1) or hematopoietic stem cells (CD34 and CD45). Cont: isotype control. (**C**) After ADSCs were cultured under osteogenic inductive conditions for 21 days, mineralized nodules were detected following alizarin red staining. ADSCs formed lipid clusters that stained positive for Oil Red O after 21 days of adipogenic induction. Scale bars represent 100 µm.

To identify putative stem cells, single-cell suspensions were generated from rat adipose tissues. Colonies that stained with toluidine blue exhibited typical fibroblastic morphology. These cells were termed clonogenic populations of ADSCs (Fig. 1A). *Ex vivo* expanded ADSCs expressed the cell surface molecules STRO-1 (24.7%) and CD146 (31.2%), two early mesenchymal stem-cell markers. FACS profiling also showed that ADSCs expressed CD31 (1.8%), CD34 (2.4%), CD45 (2.0%), CD90 (100%), and CD105 (96.1%) (Fig. 1B).The potential for osteoblastic and adipogenic differentiation of ADSCs were determined. The ADSCs were grown in osteogenic-inducing medium; dark red mineralized bone matrix (bone nodules) was visualized in alizarin red-stained sections (Fig. 1C). Adipogenic differentiation of ADSCs was confirmed by Oil Red O staining. The induced ADSCs populations formed Oil Red O-positive lipid clusters after 3 weeks of adipogenic induction (Fig. 1C).

### ADSCs acquired cementoblast features after DFC-CM treatment

**Figure 2:**
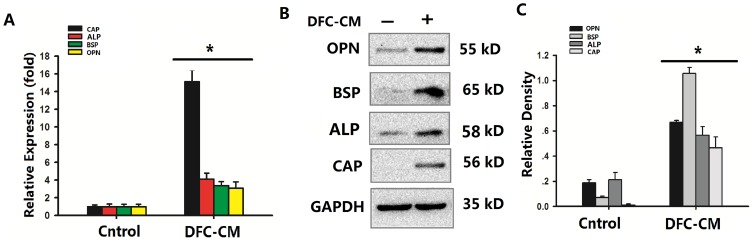
DFC-CM microenvironments induce ADSC differentiation into cementoblast-like cells. (**A**) Real-time PCR analysis of the expression of CAP, ALP, BSP and OPN in ADSCs grown in DFC-CM for 7 days. The expression levels were normalized to that of β-actin. The results represent mean values (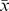 ± SD) from three independent experiments performed in triplicates. *P<0.05 vs. the control group. (**B**) CAP, BSP, and OPN were measured by Western blot in ADSCs grown in basal medium or in cells cultured in DFC-CM for 7 d. GAPDH was used as control for equal loading. (**C**) Quantitative analysis of the OPN, BSP, ALP and CAP. *P<0.05 vs. the control group.

ADSCs have been reported to possess the potential to differentiate into cementoblasts [8]. The attachment proteins CAP, ALP, BSP, and OPN act as cementum components, and their presence appears to be limited to cementoblasts and their progenitors [25], [26]. After 7 days of DFC-CM treatment the mineralization behavior of differentiated ADSCs was observed, in which mineralization-related markers, including CAP, ALP, BSP, and OPN, were detected at mRNA or protein levels. The expression of these marker genes in ADSCs treated with DFC-CM compared to the control group increased by the factor 15.1247 (CAP), 4.0856 (ALP), 3.3543 (BSP) and 3.1054 (OPN), respectively (Fig. 2A). To further confirm these findings, a Western blot analysis was used for protein detection. The results also indicated that CAP, BSP, and OPN protein levels increased in ADSCs treated with DFC-CM for 7 days (Fig. 2B). CAP proteins were expressed only when cells were grown in the presence of DFC-CM (Fig. 2B).The histogram showed the quantitative analysis of the OPN, BSP, ALP and CAP. Significantly higher expression of the proteins were again observed in the DFC-CM induced group than the control group (Fig. 2C).

### DCF-CM microenvironment suppressed Wnt/β-catenin signaling pathway of ADSCs

**Figure 3:**
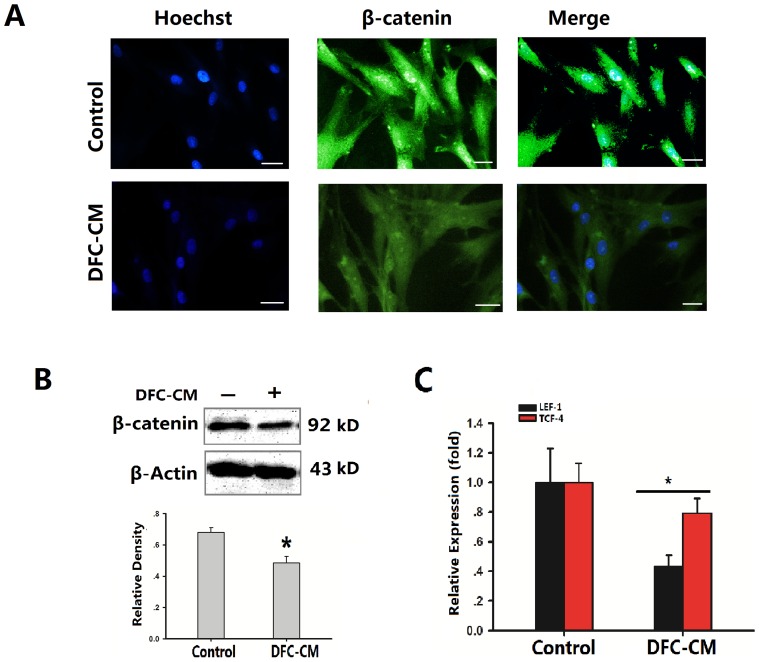
DFC-CM condition effect Wnt/β-catenin signaling pathway in ADSCs. (**A**) Immunocytochemical staining showed that ADSCs cultured in basic medium or in DFC-CM condition expressed β-catenin. Scale bar represents 100 µm. (**B**) Beta-Catenin levels were examined by Western blot analysis and scanning densitometer. Beta-actin was used as internal control. (**C**). LEF-1and TCF-4 mRNA were subjected to real time-PCR analysis after cells were cultured in DFC-CM or basal medium (control) for 7 days. The expression levels were normalized to those of β-actin. The results represent mean values (SD) from three independent experiments performed in triplicates. *P<0.05 vs. the control group.

Wnt/β-catenin signaling plays a critical role in bone formation and regeneration [9], [23]. Dentin and cementum share many similarities with bone in their biochemical compositions and biomechanical properties. During tooth development, it was found that canonical Wnt signaling operates at multiple stages of tooth morphogenesis [27]. Several Wnt genes are broadly expressed in dental epithelium and mesenchyme during early tooth development [28]. Thus, we postulated that the DFC-CM cultured condition would affect Wnt/β-catenin signaling pathway during the differentiation process of ADSCs. In order to investigate of the mechanism of the Wnt/β-catenin signaling pathway on cementum regeneration, immunofluorescence staining and Western Blot were used for determining the protein expression in ADSCs cultured in DFC-CM. The results showed that intense staining of β-catenin protein was observed in both the control and the DFC-CM cultured groups, while the positive expression rate decreased in the DCF-CM treated group (Fig. 3A). Western blot analysis confirmed that DFC-CM induction of ADSCs could significantly decrease β-catenin expression levels (P<0.05, Fig. 3B). Canonical Wnt signaling stabilizes β-catenin, which subsequently translocates into the nucleus to activate the transcription of TCF/LEF family genes. Consistent with the effects of LEF/TCF, we examined the mRNA expression of LEF-1 and TCF-4. The results showed that LEF-1 and TCF-4 expression levels were significantly decreased under DFC-CM condition compared to the control group (Fig. 3C).

### *In vitro* regulation of ADSCs differentiation into cementoblast-like cells by the Wnt/β-catenin signaling pathway

Canonical Wnt signaling appears to be capable of maintaining certain stem cell populations in a proliferative and non-differentiating state. The inhibition of canonical Wnt signaling either by deleting LEF-1 or by over-expressing the Wnt antagonist Dickkopf (DKK)1 arrests tooth morphogenesis at the late bud stage [29], [30], and oral epithelium expressing constitutively active β-catenin results in the formation of multiple teeth, following transplantation to a kidney capsule [31]. To investigate whether the differentiation potential of ADSCs cultured in DFC-CM could be improved, we cultured ADSCs in the presence of exogenous recombinant rat Wnt3a or DKK1 to activate or inactivate the Wnt/β-catenin signaling pathway.

**Figure 4:**
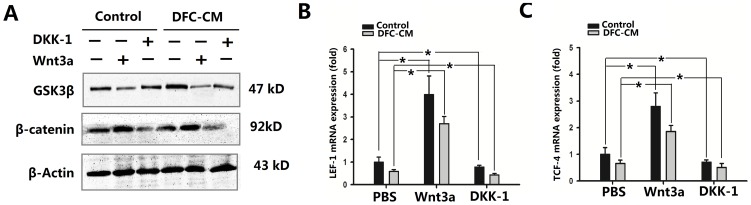
Wnt3a or DKK1 activate or inactivate the Wnt/β-catenin signaling pathway. (**A**) The expression of β-catenin as well as GSK3β were measured by Western blot in ADSCs grown in DFC-CM or in basal medium (control) with or without the presence of Wnt3a (100 ng/mL)/DKK-1 (100 ng/mL) for 7 d. Beta-actin was used as control for equal loading. (**B, C**) Expression of LEF-1 (**B**) and TCF-4 (**C**) genes in ADSCs cultured in basal medium or in DFC-CM with Wnt3a (100 ng/mL)/DKK-1 (100 ng/mL) for 7 d. PBS was used as control condition. The expression levels were normalized to that of β-actin. The results represent mean values (SD) from three independent experiments performed in triplicates. *P<0.05 vs. the PBS group.

Firstly, we determined the levels of β-catenin and GSK3β (glycogen synthase kinase 3 beta) by Western blot analysis during a period of 7 d in order to investigate whether treatment of ADSCs with recombinant rat Wnt3a (100 ng/mL) influenced the Wnt signaling pathway in ADSCs on the protein level. ADSCs expressed the β-catenin protein in both the control group and the DCM-CM cultured group, and its expression was increased following Wnt3a treatment (Fig. 4A). In marked contrast, treatment with DKK-1 at 100 ng/mL resulted in decreased expression levels of β-catenin, with the strongest decrease observed in the group of ADSCs grown in DFC-CM in the presence of DKK-1 (Fig. 4A). After cultured in DFC-CM, the expression of GSK3β was up-regulated in ADSCs. We found that treatment with Wnt3a led to decreased expression of GSK3β in both the control group and the DFC-CM group. Moreover, when we inhibited the Wnt pathway with DKK-1 under control or DFC-CM induction conditions, GSK3β expression was slightly changed (Fig. 4A). In addition, our results showed that expression of LEF-1 and TCF-4 in both the control groups and the DFC-CM induction groups were up-regulated in the presence of exogenous DKK1 and down-regulated in the presence of exogenous Wnt3a (Fig. 4B, C).

**Figure 5:**
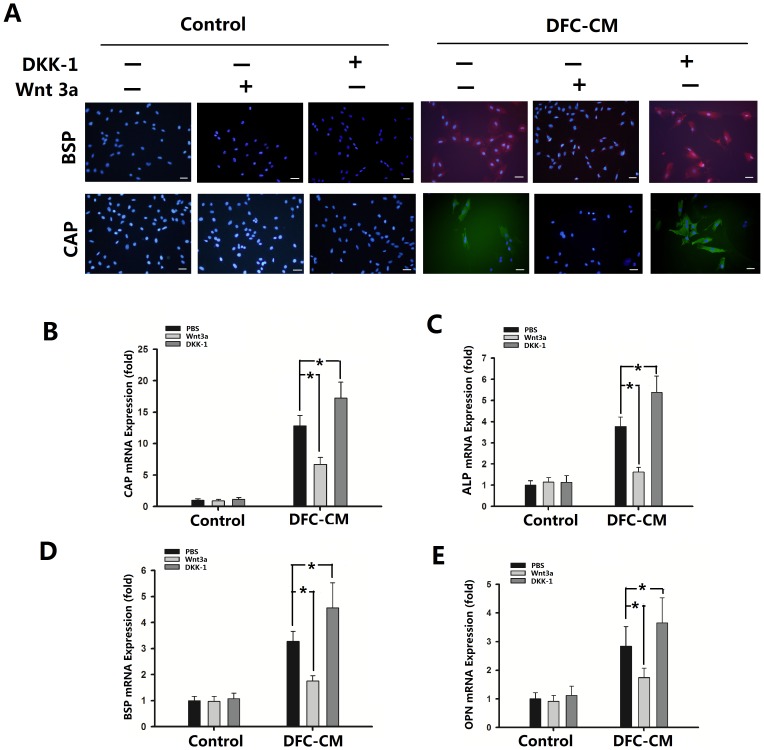
Regulation of cementogenic differentiation of ADSCs by Wnt/β-catenin signaling pathway. (**A**) Immunostaining of BSP and CAP in ADSCs cultured in basal medium or DFC-CM with or without Wnt3a (100 ng/mL)/DKK-1 (100 ng/mL) treatment for 7 d. Scale bar represents 100 µm. (**B–E**) Expression of CAP (**B**), ALP (**C**), BSP (**D**), and OPN (**E**) genes in ADSCs cultured in basal medium or in DFC-CM with Wnt3a (100 ng/mL)/DKK-1 (100 ng/mL) for 7 d. PBS was used as control condition. The expression levels were normalized to that of β-actin. The results represent mean values (SD) from three independent experiments performed in triplicates. *P<0.05 vs. the PBS group.

In order to investigate the ability of ADSCs to differentiate into cementoblast-like cells, immunofluorescence staining was used for determining the protein expression in ADSCs. The results showed that ADSCs cultured in DFC-CM were positive for BSP and CAP. And then we treated ADSCs with DKK-1(100 ng/mL) under DFC-CM induction conditions, we found that the number of BSP and CAP positive expression cells were increased (Fig. 5A). Furthermore, Wnt3a treatment showed BSP and CAP negative expression at both the control group and the DFC-CM group (Fig. 5A). DKK-1 significantly promoted ADSC differentiation into cementoblast-like cells as shown by Real Time-PCR. Expression levels of CAP (Fig. 5B), ALP (Fig. 5C), BSP (Fig. 5D) and OPN (Fig. 5E) in DFC-CM groups were significantly up-regulated compared with DFC-CM+PBS group. In contrast to treatment with DKK-1, Wnt3a treatment significantly down-regulated the expression of CAP (Fig. 5B), ALP (Fig. 5C), BSP (Fig. 5D) and OPN (Fig. 5E) groups compared with the DFC-CM+PBS group. The same passage of ADSCs and the same conditions were used for the experiments showing up-regulation of the Wnt pathway by Wnt3a and down-regulation of the Wnt/β-catenin pathway by DKK-1.

## Discussion

It is widely accepted that periodontal defects usually do not heal spontaneously. Periodontal regeneration requires new connective tissue attachment to the root surface, a process that involves the formation of new cementum on previously exposed root surfaces, the synthesis of Sharpey's fibers and their insertion into newly formed cementum. Therefore, a variety of procedures have been advocated in order to ensure an outcome in which periodontal tissue regeneration can occur. Many studies have reported that dental stem cells can form structures resembling tooth tissue, and that dental pulp stem cells (DPSCs) and dental follicle cells have the ability to form odontoblast-like cells that produce dentine [32]. However, the paucity of supply of a patients' own dental stem cells limits the use of this approach. Stem cells are uncommitted entities capable of both self-renewal and differentiation into multiple cell lineages. A recent study showed that ADSCs are defined as non-hematopoietic cells (CD45^−^, CD34^−^) but express other molecules, the combination of which is largely used for their description: CD73^+^, CD44^+^, CD105^+^, CD90^+^, CD146^+^ and STRO-1^+^ [33]. We also found that ADSCs have the potential to differentiate into osteogenic and adipogenic cell lineages. ADSCs represent a very promising potential for tissue regeneration.

Cementogenesis has received attention due to its importance in periodontal maintenance and regeneration. In the present study, expression of CAP as a putative cementoblast marker has been detected in cultured ADSCs stimulated with DFC-CM. In addition to osteoblastic/cementoblastic differentiation of ADSCs, the osteogenic differentiation of PDLSC(periodontal ligament stem cells) and DPSC(dental pulp stem cell) has been also reported. PDLSCs were allowed to differentiate in the presence of ascorbic acid-2-phosphate. This resulted in the expression of alkaline phosphatase, mineralization-related genes, and the formation of mineralized nodules, thus indicating osteoblastic/cementoblastic cell lineage differentiation [22]. In another study, Ikeda et al. reported *in vitro* osteoblastic/cementoblastic differentiation of DPPSCs in the presence of ascorbic acid, b-glycerophosphate, and dexamethasone. This differentiation was evidenced by high alkaline phosphatase activity, osteocalcin content, and mineralized nodule formation [34].

The dental follicle (DF) is a specialized connective tissue, a loose connective tissue sac that surrounds the unerupted tooth and which plays different roles in the development of a tooth. Although some studies suggest that some of the cementoblasts may arise from Hertwig's epithelial root sheath, as well as from the DF [35], other studies indicate that all cementoblasts arise from the DF [36]. Together with our results that a DFC-CM microenvironment could induce ADSC differentiation into cementoblast-like cells, these findings suggest that the dental follicle could be a niche for stem cells.

Persistent stabilization of β-catenin in the dental mesenchyme leads to premature differentiation of odontoblasts and differentiation of cementoblasts, and induces excessive dentin and cementum formation *in vivo* [37]. Intriguingly, our results showed that the β-catenin levels were lower in ADSCs cultured in DFC-CM than in those cultured in basal medium. β-catenin is known to associate with the LEF/TCF transcription factor family and promote the expression of several genes through the recruitment of other factors to form a transcriptionally active complex [9]–[11]. LEF-1 is reported to have an important role in osteoblast maturation for its ability in the regulation of expression of genes involved in the stimulation of bone formation, such as Runx2 and Col-1 [38], [39]. A recent report examining the role of the LEF-1 in the regulation of bone mass found that LEF1+/− female mice had decreased trabecular bone mass due to reduced osteoblast activity [40]. TCF4 is expressed in the ventral diencephalon early in pituitary development, rostral to a domain of BMP and FGF expression [41]. β-catenin/TCF-4 transcriptional complex and its downstream targe c-Myc are both regulating the OPN expression [42]. In our study, DFC-CM treatment resulted in significantly decreased levels of LEF-1 and TCF-4 in ADSCs. This is mainly due to the fact that the Wnt target genes are kept in a repressed state in the nucleus by interacting with TCF and LEF transcription factors with associated co-repressors [43]. Canonical Wnt signaling has been reported to contribute positively to osteoblast differentiation as measured by induced expression of genes encoding ALP and Runx2 in murine pluripotent mesenchymal cell lines, C3H10T1/2, C2C12, or in MC3T3-E1, a murine osteoblast cell line [44], [45]. On the other hand, negative effects of canonical Wnt signaling on osteoblast differentiation have also been reported. Activation of Wnt signaling by Wnt3a or LiCl inhibits dexamethasone-induced mineralized nodule formation with reduced osteogenic markers such as the ALP gene in human mesenchymal cells and C3H10T1/2 [24], [46], [47]. In functional studies, Chen et al. used inducible conditional mutations of β-catenin in mice [48]. After fracture and deletion of β-catenin, MSCs could be attracted and could proliferate, but they differentiated into chondroblasts instead of osteoblasts, leading to failed bone healing.

The aim of this research was to successfully regenerate cementum tissue. Dkk1 specifically inhibits canonical Wnt signaling by binding as a high-affinity antagonist to LRP5/6 co-receptors. Currently, we do not know whether down-regulated β-catenin levels could help to promote ADSC differentiation into cementoblast-like cells via inhibition of the n Wnt/β-catenin signaling pathway *in vitro*. We found that ADSCs treated with DKK-1 displayed dramatically reduced baseline levels of β-catenin, whereas DKK-1 induced CAP, ALP, BSP, and OPN activation to promote osteoblastic/cementoblastic differentiation. Enhanced Wnt/β-catenin signaling either by Wnt overexpression or by deficiency of Wnt antagonists is associated with increased bone formation in mice and humans [9], [20]. However, this mechanism is not consistent with our results, along with those of recent studies [18], [23], [24] that activation of the canonical Wnt pathway inhibited osteogenic differentiation of ADSCs in DFC-CM induction microenvironments. Here, we provide evidence that Wnt/β-catenin up-regulated by 100 ng/mL Wnt3a significantly decreased mRNA expression levels of CAP, ALP, BSP, and OPN in ADSCs. At this point, the specific signaling pathway(s) involved in DKK or Wnt3a-induced ADSCs differentiation remain unclear.

In this study, it has been suggested that the use of ADSCs could be an alternative potent source for the application of tissue engineering given their comparatively easy accessibility and multidifferentiation capacity. Our study has provided new insights into the molecular mechanisms of the Wnt/β-catenin signaling pathway, thus showing that it acts as an important regulator controlling cementoblastic differentiation of ADSCs induced in a DFC-CM microenvironment. A down-regulated Wnt/β-catenin signaling pathway could promote ADSC differentiation into cementoblast-like cells in a DFC-CM microenvironment. Future efforts should focus on developing an in-depth understanding of the regulation and functions of the Wnt pathway in the cementoblastic differentiation of MSCs, which could improve periodontal tissue regeneration.

## References

[1] Pihlstrom BL, Michalowicz BS, Johnson NW (2005) Periodontal diseases. Lancet 366: 1809–1820. 16298220 10.1016/S0140-6736(05)67728-8

[2] Cochran DL, Wozney JM (1999) Biological mediators for periodontal regeneration. Periodontol 2000 19: 40–58. 10321215 10.1111/j.1600-0757.1999.tb00146.x

[3] Liu S, Zhang H, Zhang X, Lu W, Huang X, et al (2011) Synergistic angiogenesis promoting effects of extracellular matrix scaffoldsand adipose-derived stem cells during wound repair. Tissue Eng Part A 17: 725–739. 20929282 10.1089/ten.TEA.2010.0331

[4] Shiraishi T, Sumita Y, Wakamastu Y, Nagai K, Asahina I (2012) Formation of engineered bone with adipose stromal cells from buccal fat pad. J Dent Res 91: 592–597. 22538411 10.1177/0022034512445633

[5] Declercq HA, De Caluwé T, Krysko O, Bachert C, Cornelissen MJ (2013) Bone grafts engineered from human adipose-derived stem cells in dynamic 3D-environments. Biomaterials 34: 1004–1017. 23146435 10.1016/j.biomaterials.2012.10.051

[6] Bartold PM, Shi S, Gronthos S (2006) Stem cells and periodontal regeneration. Periodontol 2000 40: 164–172. 16398692 10.1111/j.1600-0757.2005.00139.x

[7] Ivanovski S, Gronthos S, Shi S, Bartold PM (2006) Stem cells in the periodontal ligament. Oral Dis 12: 358–363. 16792719 10.1111/j.1601-0825.2006.01253.x

[8] Wen X, Nie X, Zhang L, Liu L, Deng M (2011) Adipose tissue-deprived stem cells acquire cementoblast features treated with dental follicle cell conditioned medium containing dentin non-collagenous proteins in vitro. Biochem Biophys Res Commun 409: 583–589. 21619870 10.1016/j.bbrc.2011.05.067

[9] De Boer J, Wang HJ, Van Blitterswijk C (2004) Effects of Wnt signaling on proliferation and differentiation of human mesenchymal stem cells. Tissue Eng 10: 393–401. 15165456 10.1089/107632704323061753

[10] Krishnan V, Bryant HU, Macdougald OA (2006) Regulation of bone mass by Wnt signaling. J Clin Invest 116: 1202–1209. 16670761 10.1172/JCI28551 PMC1451219

[11] Ling L, Nurcombe V, Cool SM (2009) Wnt signaling controls the fate of mesenchymal stem cells. Gene 433: 1–7. 19135507 10.1016/j.gene.2008.12.008

[12] Engert S, Burtscher I, Liao WP, Dulev S, Schotta G, et al (2013) Wnt/β-catenin signalling regulates Sox17 expression and is essential for organizer and endoderm formation in the mouse. Development 140: 3128–3138. 23824574 10.1242/dev.088765

[13] Kim TH, Bae CH, Lee JC, Ko SO, Yang X, et al (2013) β-catenin is required in odontoblasts for tooth root formation. J Dent Res 92: 215–221. 23345535 10.1177/0022034512470137

[14] Ouyang H, Zhuo Y, Zhang K (2013) WNT signaling in stem cell differentiation and tumor formation. J Clin Invest 123: 1422–1424. 23524963 10.1172/JCI69324 PMC3613938

[15] Zhu X, Zhao P, Liu Y, Zhang X, Fu J, et al (2013) Intra-epithelial requirement of canonical Wnt signaling for tooth morphogenesis. J Biol Chem 288: 12080–12089. 23525146 10.1074/jbc.M113.462473 PMC3636893

[16] Aurrekoetxea M, Lopez J, García P, Ibarretxe G, Unda F (2012) Enhanced Wnt/β-catenin signalling during tooth morphogenesis impedes cell differentiation and leads to alterations in the structure and mineralisation of the adult tooth. Biol Cell 104: 603–617. 22671936 10.1111/boc.201100075

[17] Ibarretxe G, Aurrekoetxea M, Crende O, Badiola I, Jimenez-Rojo L, et al (2012) Epiprofin/Sp6 regulates Wnt-BMP signaling and the establishment of cellular junctions during the bell stage of tooth development. Cell Tissue Res 350: 95–107. 22868911 10.1007/s00441-012-1459-8

[18] Scheller EL, Chang J, Wang CY (2008) Wnt/beta-catenin inhibits dental pulp stem cell differentiation. J Dent Res 87: 126–130. 18218837 10.1177/154405910808700206 PMC2906770

[19] Nemoto E, Koshikawa Y, Kanaya S, Tsuchiya M, Tamura M, et al (2009) Wnt signaling inhibits cementoblast differentiation and promotes proliferation. Bone 44: 805–812. 19442631 10.1016/j.bone.2008.12.029

[20] Cho HH, Kim YJ, Kim SJ, Kim JH, Bae YC, et al (2006) Endogenous Wnt signaling promotes proliferation and suppresses osteogenic differentiation in human adipose derived stromal cells. Tissue Eng 12: 111–121. 16499448 10.1089/ten.2006.12.111

[21] Tobita M, Uysal CA, Guo X, Hyakusoku H, Mizuno H (2013) Periodontal tissue regeneration by combined implantation of adipose tissue-derived stem cells and platelet-rich plasma in a canine model. Cytotherapy S1465–3249. 10.1016/j.jcyt.2013.05.007 23849975

[22] Seo BM, Miura M, Gronthos S, Bartold PM, Batouli S, et al (2004) Investigation of multipotent postnatal stem cells from human periodontal ligament. Lancet 364: 149–155. 15246727 10.1016/S0140-6736(04)16627-0

[23] Liu G, Vijayakumar S, Grumolato L, Arroyave R, Qiao H, et al (2009) Canonical Wnts function as potent regulators of osteogenesis by human mesenchymal stem cells. J Cell Biol 185: 67–75. 19349579 10.1083/jcb.200810137 PMC2700509

[24] Liu N, Shi S, Deng M, Tang L, Zhang G, et al (2011) High levels of β-catenin signaling reduce osteogenic differentiation of stem cells in inflammatory microenvironments through inhibition of the noncanonical Wnt pathway. J Bone Miner Res 26: 2082–2095. 21638320 10.1002/jbmr.440

[25] BarKana I, Narayanan AS, Grosskop A, Savion N, Pitaru S (2000) Cementum attachment protein enriches putative cementoblastic populations on root surfaces in vitro. J Dent Res 79: 1482–1488. 11005732 10.1177/00220345000790070901

[26] Komaki M, Iwasaki K, Arzate H, Narayanan AS, Izumi Y, et al (2012) Cementum protein 1 (CEMP1) induces a cementoblastic phenotype and reduces osteoblastic differentiation in periodontal ligament cells. J Cell Physiol 227: 649–657. 21465469 10.1002/jcp.22770

[27] Liu F, Chu EY, Watt B, Zhang Y, Gallant NM, et al (2008) Wnt/beta-catenin signaling directs multiple stages of tooth morphogenesis. Dev Biol 313: 210–224. 18022614 10.1016/j.ydbio.2007.10.016 PMC2843623

[28] Sarkar L, Sharpe PT (1999) Expression of Wnt signalling pathway genes during tooth development. Mech Dev 85: 197–200. 10415363 10.1016/s0925-4773(99)00095-7

[29] van Genderen C, Okamura RM, Fariñas I, Quo RG, Parslow TG, et al (1994) Development of several organs that require inductive epithelial-mesenchymal interactions is impaired in LEF-1-deficient mice. Genes Dev 8: 2691–2703. 7958926 10.1101/gad.8.22.2691

[30] Andl T, Reddy ST, Gaddapara T, Millar SE (2002) WNT signals are required for the initiation of hair follicle development. Dev Cell 2: 643–653. 12015971 10.1016/s1534-5807(02)00167-3

[31] Järvinen E, Salazar-Ciudad I, Birchmeier W, Taketo MM, Jernvall J, et al (2006) Continuous tooth generation in mouse is induced by activated epithelial Wnt/beta-catenin signaling. Proc Natl Acad Sci USA 103: 18627–18632. 17121988 10.1073/pnas.0607289103 PMC1693713

[32] Guo W, He Y, Zhang X, Lu W, Wang C, et al (2009) The use of dentin matrix scaffold and dental follicle cells for dentin regeneration. Biomaterials 30: 6708–6723. 19767098 10.1016/j.biomaterials.2009.08.034

[33] Jahagirdar BN, Verfaillie CM (2005) Multipotent adult progenitor cell and stem cell plasticity. Stem Cell Rev 1: 53–59. 17132875 10.1385/SCR:1:1:053

[34] Ikeda E, Hirose M, Kotobuki N, Shimaoka H, Tadokoro M, et al (2006) Osteogenic differentiation of human dental papilla mesenchymal cells. Biochem Biophy Res Commun 342: 1257–1262. 10.1016/j.bbrc.2006.02.101 16516858

[35] Zeichner-David M, Oishi K, Su Z, Zakartchenko V, Chen LS, et al (2003) Role of Hertwig's epithelial root sheath cells in tooth root development. Dev Dyn 228: 651–663. 14648842 10.1002/dvdy.10404

[36] Diekwisch TG (2001) The developmental biology of cementum. Int J Dev Biol 45: 695–706. 11669371

[37] Kim TH, Lee JY, Baek JA, Lee JC, Yang X, et al (2011) Constitutive stabilization of β-catenin in the dental mesenchyme leads to excessive dentin and cementum formation. Biochem Biophys Res Commun 412: 549–555. 21854758 10.1016/j.bbrc.2011.07.116

[38] Gaur T, Lengner CJ, Hovhannisyan H, Bhat RA, Bodine PV, et al (2005) Canonical WNT signalling promotes osteogenesis by directly stimulating RUNX2 gene expression. J Biol Chem 280 39: 33132–33140. 16043491 10.1074/jbc.M500608200

[39] Kahler RA, Yingst SM, Hoeppner LH, Jensen ED, Krawczak D, et al (2008) Collagen 11a1 is indirectly activated by lymphocyte enhancer-binding factor 1 (Lef1) and negatively regulates osteoblast maturation. Matrix Biol 27 4: 330–338. 18280717 10.1016/j.matbio.2008.01.002 PMC2431459

[40] Noh T, Gabet Y, Cogan J, Shi Y, Tank A, et al (2009) Lef1haploinsufficient mice display a low turnover and low bone mass phenotype in a gender- and age- specific manner. PLoS ONE 4 5: e5438. 19412553 10.1371/journal.pone.0005438 PMC2673053

[41] Brinkmeier ML, Potok MA, Davis SW, Camper SA (2007) TCF4 deficiency expands ventral diencephalon signaling and increases induction of pituitary progenitors. Dev Biol 311 2: 396–407. 17919533 10.1016/j.ydbio.2007.08.046 PMC2693283

[42] El-Tanani M, Platt-Higgins A, Rudland PS, Campbell FC (2004) Ets gene PEA3 cooperates with beta-catenin-Lef-1 and c-Jun in regulation of osteopontin transcription. J Biol Chem 279 20: 20794–806. 14990565 10.1074/jbc.M311131200

[43] Moon RT, Kohn AD, De Ferrari GV, Kaykas A (2004) WNT and beta-catenin signalling: diseases and therapies. Nat Rev Genet 5: 691–701. 15372092 10.1038/nrg1427

[44] Rawadi G, Vayssière B, Dunn F, Baron R, Roman-Roman S (2003) BMP-2 controls alkaline phosphatase expression and osteoblast mineralization by a Wnt autocrine loop. J Bone Miner Res 18: 1842–1853. 14584895 10.1359/jbmr.2003.18.10.1842

[45] Gaur T, Lengner CJ, Hovhannisyan H, Bhat RA, Bodine PV, et al (2005) Canonical WNT signaling promotes osteogenesis by directly stimulating Runx2 gene expression. J Biol Chem 280: 33132–33140. 16043491 10.1074/jbc.M500608200

[46] de Boer J, Siddappa R, Gaspar C, van Apeldoorn A, Fodde R, et al (2004) Wnt signaling inhibits osteogenic differentiation of human mesenchymal stem cells. Bone 34: 818–826. 15121013 10.1016/j.bone.2004.01.016

[47] van der Horst G, van der Werf SM, Farih-Sips H, van Bezooijen RL, Löwik CW, et al (2005) Downregulation of Wnt signaling by increased expression of Dickkopf-1 and -2 is a prerequisite for late-stage osteoblast differentiation of KS483 cells. J Bone Miner Res 20: 1867–1877. 16160745 10.1359/JBMR.050614

[48] Chen Y, Whetstone HC, Lin AC, Nadesan P, Wei Q, et al (2007) Beta-catenin signaling plays a disparate role in different phases of fracture repair: implications for therapy to improve bone healing. PLoS Med 4: 1216–1229. 10.1371/journal.pmed.0040249 PMC1950214 17676991

